# CXCR4 increases *in-vivo* glioma perivascular invasion, and reduces radiation induced apoptosis: A genetic knockdown study

**DOI:** 10.18632/oncotarget.13295

**Published:** 2016-11-11

**Authors:** Viveka Nand Yadav, Daniel Zamler, Gregory J. Baker, Padma Kadiyala, Anat Erdreich-Epstein, Ana C. DeCarvalho, Tom Mikkelsen, Maria G. Castro, Pedro R. Lowenstein

**Affiliations:** ^1^ Department of Neurosurgery, University of Michigan Medical School, Ann Arbor, MI 48109, USA; ^2^ Department of Cell and Developmental Biology, University of Michigan Medical School, Ann Arbor, MI 48109, USA; ^3^ Current address: Department of Systems Biology, Harvard Medical School, Boston, MA 02115, USA; ^4^ Department of Neurosurgery, at the Saban Research Institute at Children's Hospital Los Angeles, CA 90027, USA; ^5^ Departments of Neurology and Neurosurgery, Henry Ford Hospital, Detroit, MI 48202, USA

**Keywords:** CXCR4 knockdown, autovascularization, perivascular invasion, glioma radiotherapy resistance, combination therapies

## Abstract

Glioblastoma (GBM) is a highly invasive brain tumor. Perivascular invasion, autovascularization and vascular co-option occur throughout the disease and lead to tumor invasion and progression. The molecular basis for perivascular invasion, i.e., the interaction of glioma tumor cells with endothelial cells is not well characterized. Recent studies indicate that glioma cells have increased expression of CXCR4. We investigated the *in-vivo* role of CXCR4 in perivascular invasion of glioma cells using shRNA-mediated knock down of CXCR4. We show that primary cultures of human glioma stem cells HF2303 and mouse glioma GL26-Cit cells exhibit significant migration towards human (HBMVE) and mouse (MBVE) brain microvascular endothelial cells. Blocking CXCR4 on tumor cells with AMD3100 *in-vitro*, inhibits migration of GL26-Cit and HF2303 toward MBVE and HBMVE cells. Additionally, genetic down regulation of CXCR4 in mouse glioma GL26-Cit cells inhibits their *in-vitro* migration towards MBVE cells; in an *in-vivo* intracranial mouse model, these cells display reduced tumor growth and perivascular invasion, leading to increased survival. Quantitative analysis of brain sections showed that CXCR4 knockdown tumors are less invasive. Lastly, we tested the effects of radiation on CXCR4 knock down GL26-Cit cells in an orthotopic brain tumor model. Radiation treatment increased apoptosis of CXCR4 downregulated tumor cells and prolonged median survival. In summary, our data suggest that CXCR4 signaling is critical for perivascular invasion of GBM cells and targeting this receptor makes tumors less invasive and more sensitive to radiation therapy. Combination of CXCR4 knock down and radiation treatment might improve the efficacy of GBM therapy.

## INTRODUCTION

Glioblastoma (GBM) is an aggressive brain tumor with high morbidity and mortality rates. These tumors are associated with poor prognosis due to their ability to migrate away from the central tumor and invade healthy brain tissue by growing within perivascular spaces through various mechanisms of perivascular invasion, i.e., vessel co-option and autovascularization [1–4]. Biological characteristics include high invasiveness, uncontrollable proliferation, and angiogenesis, making it resistant to currently available surgical, radiation, and chemotherapy treatments [5–9]. It is thus essential to identify the mediators promoting the growth and recurrence of these tumors.

Recently we described that throughout glioma growth invasion occurs via auto-vascularization, a mechanism related to vessel co-option, by which glioma cells invade and allow brain tumor to become vascularized by normal blood vessels. Tumor cells proliferate by utilizing perivascular space as a channel of invasion [4, 10–13]. Although the molecular mechanisms by which this process occurs remains poorly researched, targeting it could have therapeutic value. Chemokine receptors are being studied currently as therapeutic targets and CXCR4 is the most widely expressed chemokine receptor on cancer cells including breast, prostrate, pancreatic, kidney and brain cancer cells [14–16]. CXCR4 binds to its ligand CXCL12 (SDF-1) and gets activated. Recent studies note that the progress of GBM is driven by glioblastoma stem-like cells (GSCs), critical promoters of tumor growth, invasion, and neovascularization [3, 7, 8, 17, 18]. CXCR4 has been found to be upregulated in GSCs upon activation with CXCL12, a CXCR4 ligand [19]. There is evidence that disruption of CXCR4 results in a reduction of GSC markers and reduction in tumor cells proliferation [20–22]. Additionally, it has been found that radiation therapy triggers the upregulation of hypoxia-inducible factor 1 (HIF-1) and CXCR4. HIF-1 consequently induces tumor secretion of CXCL12, which binds to CXCR4 on proangiogenic bone marrow–derived cells (BMDCs), recruiting them to become endothelial cells within the tumor [23, 24]. CXCL12 released in the sub-ventricular zones of the brain offer GBM resistance to radiation and targeting the CXCL12/CXCR4 signaling system sensitizes SVZ-nested GBM cells to radiation [25]. Thus, the inhibition of CXCR4 (and its interaction with CXCL12) is a potential target for inhibiting glioma cell invasion and recurrence. Hence, this study explores the effects of C-X-C Chemokine Receptor 4, CXCR4 and its role in perivascular Invasion.

In this study, we explore the crucial role of CXCR4 in the invasion of tumor cells into the perivascular space by down-regulating the expression of CXCR4. We hypothesized that knocking down CXCR4 will decrease perivascular invasion of tumor cells. Furthermore, by treating CXCR4 knockdown tumors with radiation we aimed to increase mice's overall median survival. Although, there are reports about targeting CXCR4 in combination with current treatment regimens available for GBM patientsnone have utilized the shRNA mediated inhibition of the CXCR4 gene on glioma cells in order to understand its *in-vivo* role in glioma's perivascular invasion [26–28]. Studies use CXCR4 pharmacological inhibitors to block CXCR4 singling to achieve increased median survival in xenograft models [28–30]. However, these inhibitors have the possibility of non-specifically targeting other molecules, noting that AMD 3100 has recently been reported to be non-specific [31–35]. We studied the potential of combining radiation therapy with targeting CXCR4 by knocking down the gene with shRNA within the tumor cells. Our findings demonstrate knocking down CXCR4 significantly increases mice's overall median survival, reduces tumor migration and invasiveness along brain endothelial cells and increases the sensitivity of tumor cells to radiation therapy. Thus we propose that combined therapy of targeting CXCR4 signaling along with radiation could be a potential therapeutic strategy for the treatment of GBM.

## RESULTS

### Rodent and human brain-derived endothelial cells promote migration of mouse and human GBM tumor cells

**Figure 1:**
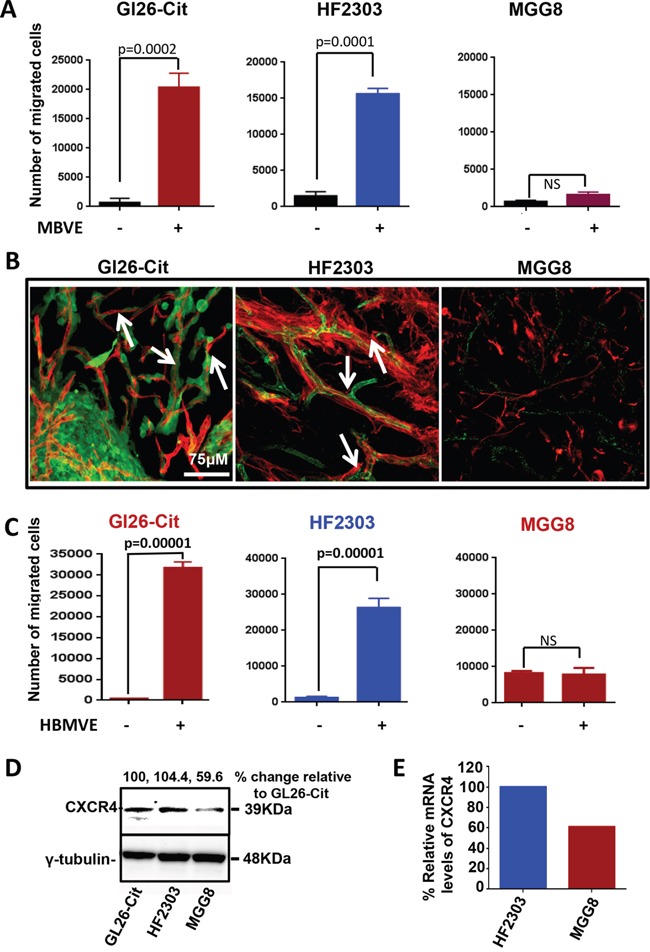
Brain-endothelial cells induce migration of GBM tumor cells **A.** Migration of mouse GL26-Cit human stem cells HF2303 and human MGG8 cell lines in response to factors secreted by mouse brain endothelial cells (MBVE) in the transwell migration assay. GL26-Cit cells showed 50 fold increase migration in response to MBVE cells (***, p= 0.0002; unpaired, two-tailed, Student t test). MBVE cells induce 7.6 fold increase migration of primary human glioma stem cell line HF2303 (***, p= 0.0002; unpaired, two-tailed, Student t test). MGG8 human GBM cells do not display migration in response to MBVE cells (ns). **B.** Fluorescence scanning confocal micrographs of, GL26-Cit, HF2303 and MGG8 cells post-tumor implantation into RAG1^−/−^ mice brain. GL26-Cit and HF2303 gliomas (green) are associated with brain micro vessels labeled with anti-CD31 antibodies (red) however not MGG8 cells. White arrowheads indicate several examples of microvasculature-associated tumor invasion. **C.** Migration of mouse GL26-Cit human stem cells HF2303 and human MGG8 cell lines in response to factors secreted by human brain endothelial cells (HBMVE) in a traswell migration assay. Similar migration as (A) is followed by tumor cells in response to HBMVE. **D.** Western blot analysis for CXCR4 expression in mouse GL26-Cit, human HF2303 and MGG8 cells. **E.** Micro-array analysis depicting mRNA levels of CXCR4 within HF2303 and MGG8 cells, Data were normalized considering HF2303 cells mRNA level as 100%.

In brain tumors, glioma cells diffusely invade the brain by active cell migration either along blood vessels, intra-parenchymally, or along white matter tracts. Molecular determinants that attract glioma cells towards blood vessels and the perivascular space are poorly understood. We have recently described that different GBM cell lines from mouse, rat and human GBM derived glioma stem cells display a specific attraction towards blood vessels *in-vivo* (Baker et al, 2014). In an effort to better understand the mechanism involved in the *in-vivo* migration of glioma cells along the blood vessels, we first tested the ability of mouse (MBVE) or human (HBMVE) brain microvessel endothelial cells to stimulate the migration of mouse and human glioma cell lines using the *in-vitro* transwell migration assay. Among different primary glioma cell lines, mouse glioma GL26-Cit and human HF2303 GBM cancer stem-cells, showed significant directional migration towards MBVE while another human GBM cell line, MGG8, did not exhibit directional migration (Figure 1A).

To examine the *in-vivo* invasion pattern of GL26-Cit, HF2303 and MGG8 cells in mouse brain, we implanted 30,000 cells of each cell line into the striatum of RAG1^−/−^ mice (N=15). Mice were euthanized at early time point which is 7 days post implantation and brains were analyzed for tumor growth. Tumor cells of GL26-Cit tumor bearing mice fluoresced green and microvessels were labeled with blood vessel-specific anti-CD31 antibodies (i.e. anti-PECAM-1). Brain tissue sections from HF2303 and MGG8 implanted mice were co-immunolabeled with antibodies against human-specific Nestin to label the tumor, and CD31 to label brain microvasculature. Confocal microscopy imaging revealed that GL26-Cit and HF2303 cells were associated with the blood vessels at the invasive border. Although MGG8 cells migrate and form tumor *in-vivo*, they did not grow along the blood vessels and failed to show any association with the brain vasculature (Figure 1B). Because HF2303 cells showed association with blood vessels, we tested whether human brain-derived microvessel endothelial cells (HBMVE) also promote migration of tumor cells when used as attractant cells in an *in-vitro* transwell migration assay. The results indicated that HBMVE cells significantly promote the migration of GL26-Cit and HF2303 cells (Figure 1C) but failed to induce migration of MGG8 cells which was similar to the response of MGG8 cells to MBVE cells. *In-vivo* data (Figure 1B) also indicated that MGG8 cells do not invade through blood vessel association. Together, the results from *in-vitro* transwell migration of GL26-Cit, HF2303 and MGG8 towards MBVE or HBMVE cells were in line with our *in-vivo* data wherein HF2303 and GL26-Cit, but not MGG8 cells, showed invasion along blood vessels. We next compared the level of CXCR4 expression in GL26-Cit, HF2303 and in MGG8 cells using Western blot analysis. Both GL26-Cit and HF2303 cell lines showed comparable levels of CXCR4 expression but MGG8 cells express lower level of CXCR4 compared to HF2303. (Figure 1D). Microarray data analysis also revealed that MGG8 cells express approximately 40 % less CXCR4 compared to HF2303 (Figure 1E). Importantly, expression level of CXCR4 correlates with the ability of GL26-Cit, HF2303 and MGG8 cells to migrate towards MBVE or HBMVE cells *in-vitro* as well as their ability to invade in perivascular space *in-vivo*.

### AMD3100 inhibits the migration of GL26-Cit and HF2303 towards mouse and human brain-derived endothelial cells. The effect of AMD3100 is stronger on rodent, than on human brain derived endothelial cells

**Figure 2:**
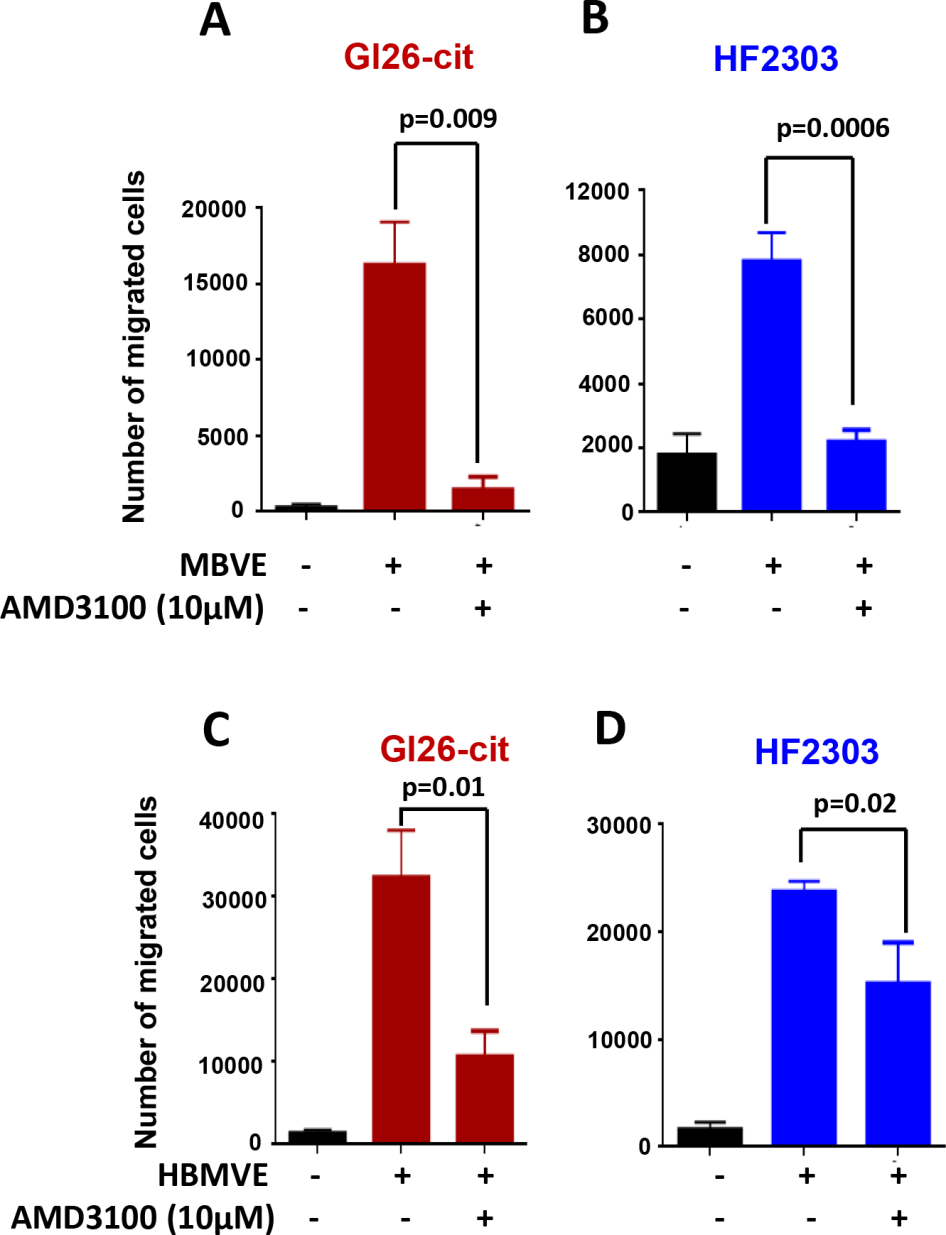
Blocking CXCR4 with AMD3100 inhibits migration of mouse and human glioma cells toward endothelial cells **A.** Migration of GL26-Cit glioma cells towards MBVE cells is inhibited in the presence of AMD3100 in a transwell migration assay (***, p=0.009). **B.** Migration of primary human glioma stem cell line HF2303 towards MBVE cells is inhibited in the presence of AMD3100 in a transwell migration assay (***, p=0006) **C.** Transwell migration assay demonstrating that the Human brain microvasculature endothelial (HBMVE) cells stimulate migration of GL26-Cit (***, p=0.00001), and primary human glioma stem cell line HF2303 (***, p=0.00001), but failed to induced migration of MGG8 cells. **D.** The migration of GL26-Cit (**, p=0.01) and HF2303 cells towards HBMVE cells was inhibited by AMD3100 (**, p=0.02).

The CXCR4-CXCL12 axis is involved in survival, migration and proliferation of various types of tumor cells, including GBM cells. To decipher the mechanism of GBM cell migration towards MBVE cells, we evaluated the involvement of CXCR4 mediated chemotactic migration of GL26-Cit and HF2303 cells towards MBVE or HBMVE cells using *in-vitro* migration assays. Tumor cells were pre-incubated with 10 μM of AMD3100 for an hour and then added to the upper chamber of the transwell. We observed that the migration of GL26-Cit and HF2303 cells towards MBVE cells were significantly inhibited by AMD3100 (Figure 2A and 2B). Further, migration of GL26-Cit and HF2303 towards HBMVE cells was also significantly inhibited by AMD3100 (Figure 2C and 2D). Data indicate that migration of GL26-Cit and HF2303 towards endothelial cells depends on chemotaxis activation of CXCR4-CXCL12 pathway.

### CXCR4 knockdown in mouse GL26-Cit cells reduces their migration capability towards MBVE cells

**Figure 3:**
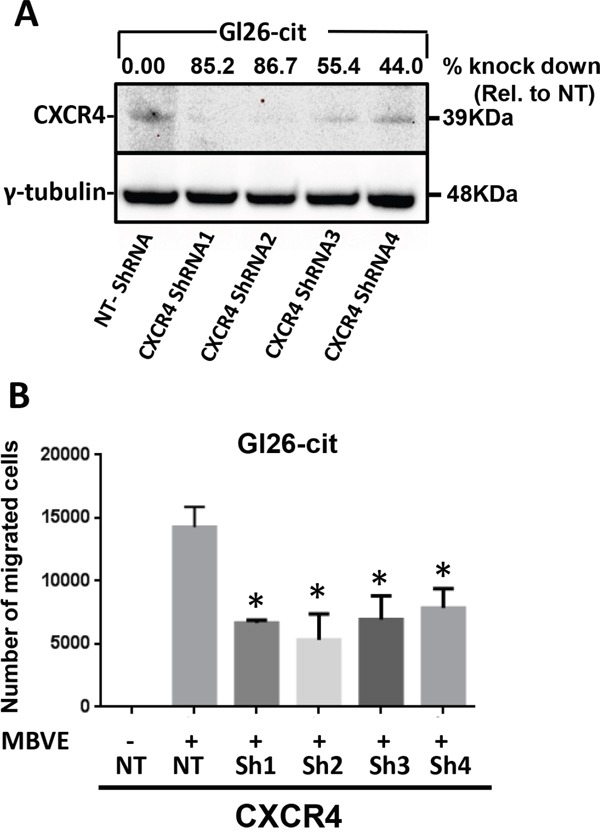
ShRNA-mediated knockdown of CXCR4 in mouse GL26-Cit glioma cells suppress their migration towards endothelial cells **A.** Western blot analysis for CXCR4 expression in GL26-Cit cells infected with lentirvirus encoding non-target shRNA (Gl26-Cit-NT) or four different short hairpin RNA directed against CXCR4 (CXCR4 shRNA). GL26-Cit cells transduced with different shRNA were stably selected and assessed for CXCR4 expression (upper band) and ϒ-tubulin (lower band) used as control. Constructs for CXCR4shRNA1 and CXCR4shRNA2 showed the highest knock down efficiency of 85% and 86.77% respectively. **B.** Migration ability of CXCR4 knockdown GL26-Cit cells towards MBVE was significantly reduced compared to control shRNA treated cells (NT) measured in transwell migration assay (***, p <0.05, student t-test).

Although AMD3100 is a clinically well accepted drug to use against CXCR4, the off target effect of AMD3100 has never been well documented. We aimed to further confirm the role of CXCR4 in GBM cell migration by knocking this gene down using commercially available CXCR4–specific shRNA hairpins from the mission shRNA (sigma). We stably selected 4 different CXCR4 shRNA hairpins transduced GL26-Cit cells (termed as GL26-Cit-Sh1CXCR4, GL26-Cit-Sh2CXCR4, GL26-Cit-Sh3CXCR4 and GL26-Cit-Sh4CXCR4) after lentiviral-mediated gene transfer. Western blot analysis confirmed the down regulation of CXCR4 expression in GL26-Cit cells (Figure 3A). CXCR4–deficient cells were referred to as GL26-Cit-ShCXCR4. CXCR4 shRNA1 and shRNA2 treated cells exhibited 85% and 86% reduction in CXCR4 protein expression respectively, compared to the control GL26-Cit-NT cells transduced with lentiviruses expressing non-targeting shRNA (GL26-Cit-NT).

If CXCR4 signaling is necessary for migration of tumor cells towards endothelial cells, downregulation of CXCR4 on tumor cells should inhibit their migration capability. We tested the migration ability of CXCR4 down-regulated GL26-Cit cells towards MBVE cells compared to non-target control. All four CXCR4 down regulated GL26-Cit cell lines showed significantly reduced migration towards MBVE cells (Figure 3B). CXCR4 shRNA1 and shRNA2 exhibited maximum knock down of CXCR4 but GL26-Cit-sh2-CXCR4 cells showed maximum reduction in migration ability, and therefore we selected GL26-Cit-sh2-CXCR4 cell line for further experiments.

### CXCR4 knockdown reduces *in-vivo* invasiveness of mouse glioma GL26-Cit tumors

**Figure 4:**
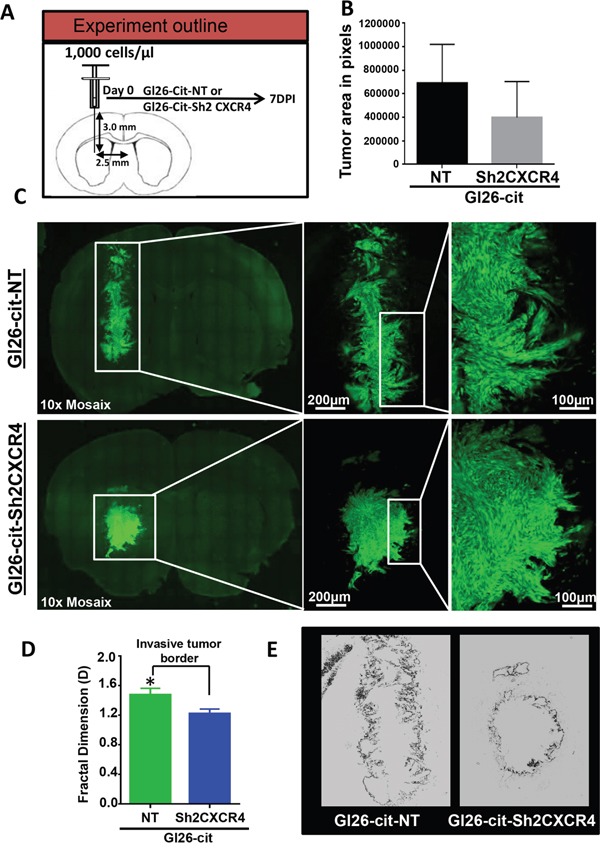
CXCR4 knockdown reduces tumor invasiveness **A.** Schematic representation for a group of NSG mice (N=4/group) implanted with GL26-Cit tumors cells with (sh2CXCR4) and without (NT) CXCR4 knockdown and perfused 7 days post implantation (dpi) to uncover tumor growth and invasiveness. **B.** Tumor size was quantified across the whole tumor from all animals with imageJ software. Tumor size of CXCR4 knockdown cells are relatively smaller compared to GL26-Cit-NT control tumor bearing mice after 7dpi. (p=0.21, ns). **C.** Coronal section of GL26-Cit-NT tumor bearing mice (top panel) reveals there is greater invasiveness at the tumor border, shown in the boxed area (higher magnification). For GL26-Cit-sh2CXCR4 tumors (bottom panel) coronal section images reveals there is reduced invasiveness at the tumor border, shown in the boxed area (higher magnification) at 7dpi. **D.** Fractal dimension quantification (D values) of tumor rims illustrate that GL26-Cit-NT are significantly more invasive compared to GL26-Cit-sh2CXCR4 tumors at 7dpi (*p <0.05, student t-test; n=4 per group). **E.** Representative fractal dimension image of tumor rims demonstrating that GL26-Cit-NT tumor have higher invasive tumor border compared to GL26-Cit-sh2CXCR4 tumor.

To examine the effect of CXCR4 knockdown on the invasion pattern of GL26-Cit tumors, we implanted 1000 GL26-Cit-Sh2CXCR4 cells or control GL26-Cit-NT cells into the striatum of NSG mice (N= 4/group) and examined the initial course of tumor progression (Figure 4A). The mice were euthanized seven days post tumor implantation and brain sections were analyzed for tumor burden, tumor growth pattern and tumor invasiveness (Figure 4). Mice implanted with GL26-Cit-NT cells formed tumors that were bigger within 7 days post-tumor implantation compared to mice with GL26-Cit-sh2CXCR4 as shown in Figure 4C. However, when we quantified tumor burden by calculating the average tumor area in both groups using ImageJ analytical software. GL26-Cit-sh2CXCR4 tumor size were not statistically different from controls (Figure 4B) (p=0.21, ns). Nevertheless, the border of GL26-Cit-NT tumors appeared more invasive compared to GL26-Cit-sh2CXCR4 tumors (Figure 4C). To determine the effect of CXCR4 down regulation on GL26-Cit cells' invasion along preexisting brain microvasculature we performed fractal dimension analysis. Our analysis indicated that invasive GL26-Cit-NT gliomas displayed an average D-value of 1.47 ± 0.015, which was significantly higher than that for GL26-Cit-sh2CXCR4 tumors (D-value=1.233 ± 0.029) (Figure 4D). Significantly low fractal dimension values of GL26-Cit-Sh2CXCR4 cells implanted mice correlates with their less invasive tumor rims (Figure 4E). Our findings show that CXCR4 down-regulated cells (GL26-Cit-sh2CXCR4) are less invasive even from early stages of tumor growth.

### Down regulation of CXCR4 in mouse glioma GL26-Cit cells prolongs survival of mice

**Figure 5:**
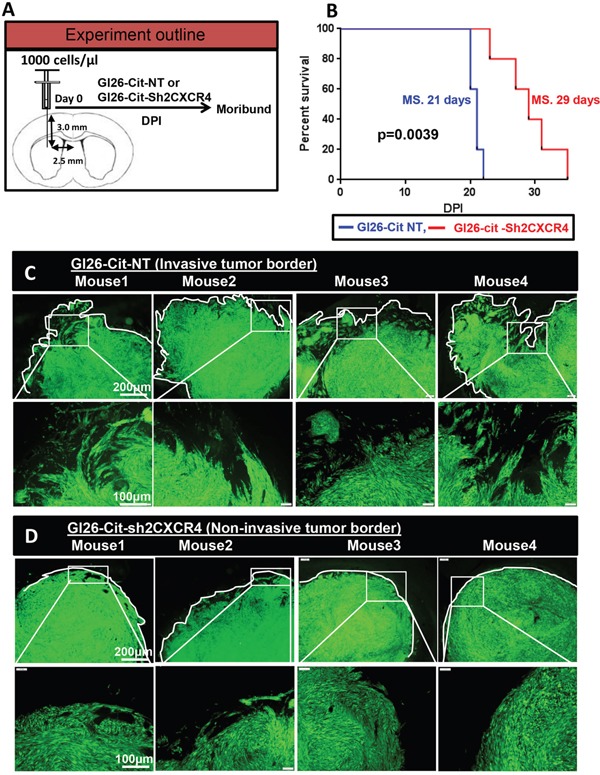
CXCR4 knockdown delays GL26-Cit tumor growth *in-vivo* **A.** Schematic representation for a group of NSG mice (N=5/group) implanted with GL26-Cit tumors with sh2CXCR4 or control shRNA for CXCR4 (NT). Mice were monitored closely during tumor progression and perfused at moribund stage. **B.** Standard Kaplan-Meier survival plot reveals notable increase in survival for mice implanted with GL26-Cit-sh2CXCR4 compared mice implanted with GL26-Cit-NT tumors (*p <0.05; n=5 per group). **C.** GL26-Cit-NT tumors budding at the tumor border illustrate infiltrative growth pattern. Top panels display low magnification images of brain sections from moribund mice with median survival of 20 days post implantation and the boxed area shown in bottom panels are a higher magnification representation of tumor border. **D.** GL26-Cit-sh2CXCR4 tumors are confined within the tumor mass illustrating non-infiltrative growth pattern. Top panels display low magnification images of brain sections from moribund mice with median survival of 26 days post implantation and the boxed area shown in bottom panels are a higher magnification representation of tumor border. Fractal dimension was quantified with imageJ software.

Our data show that inhibiting CXCR4 in glioma cells reduces the migration and invasion ability of mouse glioma GL26-Cit *in-vitro* and *in-vivo*. Therefore, we wished to test whether CXCR4 suppression would improve the median survival of mice implanted with GL26-Cit-sh2CXCR4 glioma cells. Kaplan–Meier survival analysis was carried out on NSG mice (N=10) implanted with 1000 GL26-Cit-sh2CXCR4 or GL26-Cit-NT glioma cells into the striatum (Figure 5A).

**Figure 6:**
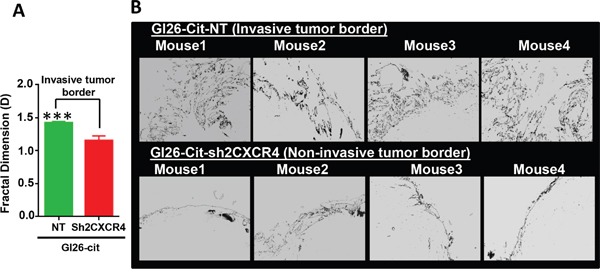
CXCR4 knockdown reduces tumor invasiveness at tumor border **A.** Fractal dimension quantification (D values) reveals that GL26-Cit-NT tumors are significantly invasive compared to GL26-Cit-sh2CXCR4 tumors (***p <0.05, student t-test; N=5 per group). **B.** Tumor rims of NT (top panel) and sh2CXCR4 (bottom panel) demonstrate that GL26-Cit-NT tumors have invasive growth pattern at the tumor border compared to GL26-Cit-shCXCR4 tumors.

Mice implanted with down regulated CXCR4 cells, GL26-Cit-sh2CXCR4, exhibited significantly prolonged survival when compared to GL26-Cit-NT mice (Figure 5B). Median survival for GL26-Cit-sh2CXCR4 tumor bearing mice was 29 days and that for GL26-Cit-NT mice was 21 days (p=0.039). The brains of these mice were sectioned and analyzed for tumor invasion (Figure 5C and 5D). Microscopic analysis revealed that mice bearing control GL26-Cit-NT cells exhibit highly invasive tumor borders in all animals as shown in (Figure 5C). On the other hand, tumor borders of mice implanted with low CXCR4 expressing GL26-Cit-sh2CXCR4 cells were less invasive (Figure 5D). To quantify tumor invasion, we performed fractal dimension analysis on tumor border of GL26-Cit-sh2CXCR4 and GL26-Cit-NT tumors using the box-counting algorithm of ImageJ quantitative analytical software (National Institutes of Health, Bethesda, MD). This allowed us to obtain a quantitative measurement of tumor invasion morphology. Tumor fractal dimension values (D values) indicated that GL26-Cit-NT gliomas displayed an average D-value of 1.424 ± 0.019. However, GL26-Cit-Sh2CXCR4 tumors had significantly lower average D-values (1.154 ± 0.071) (Figure 6A). The significantly lower fractal dimension values of GL26-Cit-Sh2CXCR4 tumors were consistent with their less invasive tumor rims as indicated with in Figure 6B, upper panel.

### Perivascular invasion of mouse glioma GL26-Cit-NT tumor cells is CXCR4 dependent

Data showing the effect of AMD3100 on tumor cells migration towards MBVE and HBMVE (Figure 2) suggest that CXCR4 plays an important role in GBM cell migration through the perivascular niche. Thus, we hypothesized that genetically modified low CXCR4-expressing tumor cells may not able to migrate through the perivascular space and will display fewer interactions with brain microvasculature.

**Figure 7:**
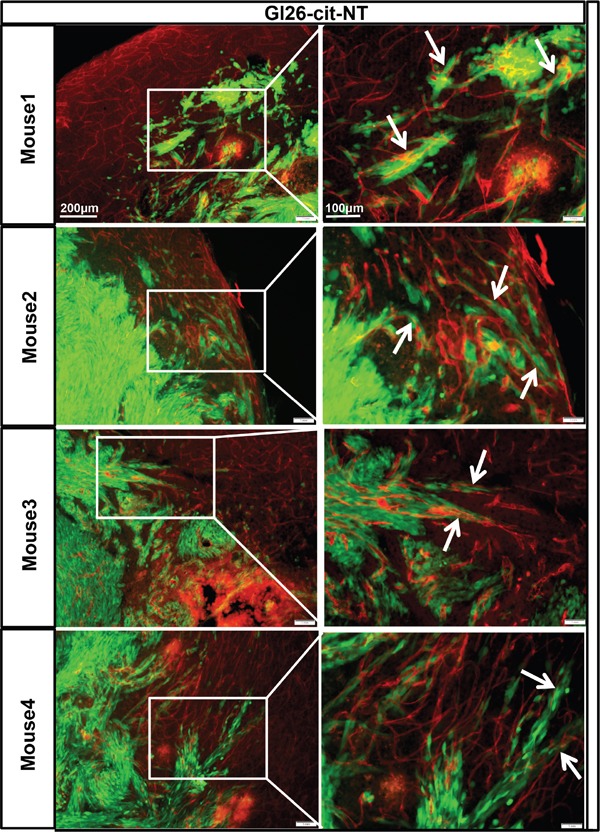
GL26-Cit tumor cells invasion along blood vessels is CXCR4 dependent GL26-Cit cells without CXCR4 knockdown (NT) expressing a phenotype for invasiveness are shown migrating away from the tumor mass towards the blood vessels within the brain parenchyma. Shown are brain sections immunohistochemistry stained for CD31 (1:500 dilution) from moribund mice implanted with GL26-Cit-NT cells on the right, and high-magnification imaging on the left for the boxed areas (for N=5 NSG mice in the study). Glioma cells are green expressing green floursent protein “citrine” and CD31 (blood vessel marker) expression is represented by Alexa-594 (red).

**Figure 8:**
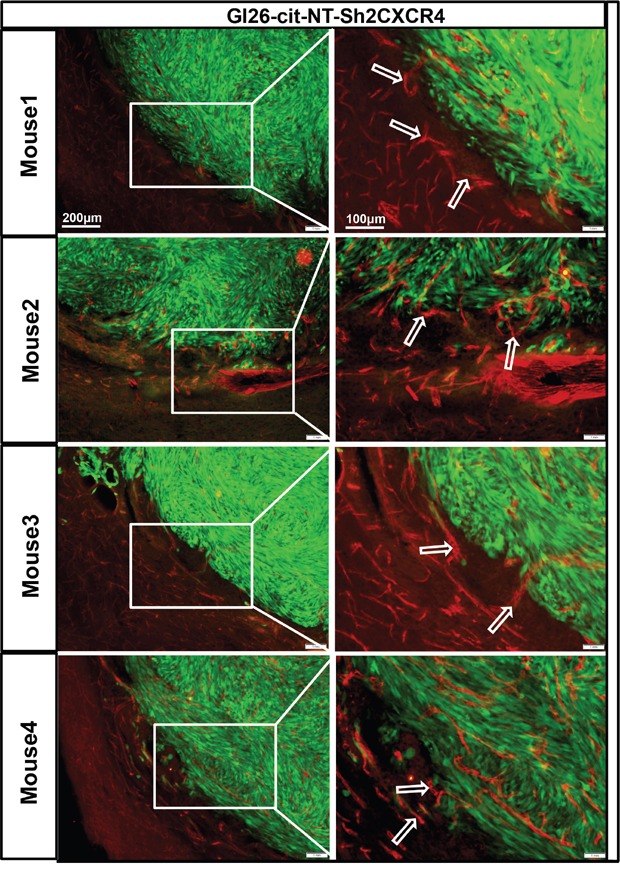
Down regulation of CXCR4 inhibits invasion of GL26-Cit tumors cells along blood vessels GL26-Cit cells with CXCR4 knockdown do not express a phenotype for invasiveness and are localized within the tumor mass. Shown are brain sections immunohistochemistry stained for CD31 (1:500 dilution) from moribund mice implanted with GL26-Cit-shCXCR4 cells on the right, and high-magnification imaging on the left for the boxed areas (for N=5 NSG mice in the study). Glioma cells are green expressing green fluorescent protein “citrine” and CD31 (blood vessel marker) expression is represented by Alexa-594 (red).

To test this hypothesis, we examined the interaction of genetically modified low CXCR4 expressing GL26-Cit-sh2CXCR4 cells versus control implanted gliomas with the existing vasculature at the tumor border. To visualize tumor cells in the brain, we implanted citrine-expressing mouse glioma cells (GL26-Cit-NT or GL26-Cit-sh2CXCR4) into the striatum of NSG mice (N=5/group) and then labeled the tumor-associated blood vessels with anti-CD31 (anti-PECAM-1)-antibody. IHC Data from GL26-Cit–NT cell implanted mice showed perivascular migration of tumor cells (as indicated in right column high power images, Figure 7) and association of tumor cells with blood vessels (indicated with white arrow heads, Figure 7). These cells did not exhibit any other known route of glioma invasion. Low CXCR4 expressing GL26-Cit-sh2CXCR4 tumor cells did not show signs of perivascular invasion (Figure 8). These findings suggest that the CXCR4-signaling aids in tumor growth and perivascular invasion, and the down regulation of CXCR4 on tumor cells abrogates their perivascular invasion.

### Radiation treatment extends survival of mice implanted with CXCR4 downregulated glioma cells

**Figure 9:**
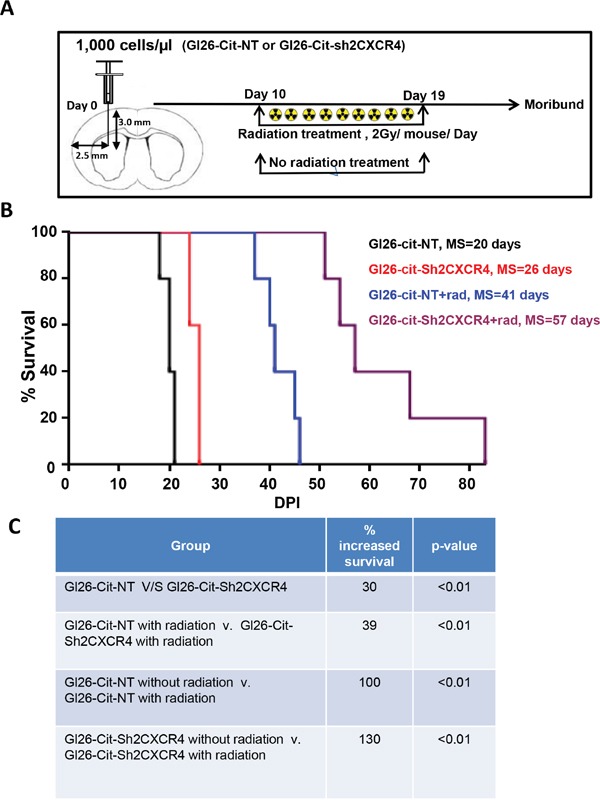
CXCR4-deficient GL26 cells are sensitive to radiation treatment **A.** Schematic depicts a group of Rag1-/- mice stereotactically injected with GL26-Cit cells with (NT) and without CXCR4 (sh2) knockdown treated after implantation from day 10 to day 19 with whole brain photon radiation. Mice were perfused with PFA at moribund state. **B.** Kaplan-Meier plot shows Rag1^−/−^ mice bearing GL26-Cit-shCXCR4 tumors survive significantly longer than the ones with GL26-Cit-NT tumors. In response to radiation treatment, mice implanted with GL26-Cit-shCXCR4 tumors exhibit an enhanced survival (Rag1^−/−^ mice, N=5 animals per group). **C.** Table comparing GL26-Cit-NT with GL26-Cit-shCXCR4, respectively, in the presence and absence of radiation treatment (** p < 0.01, log-rank test).

To determine the effect of irradiation, a treatment for malignant glioma, on CXCR4 down regulated GL26-Cit-Sh2CXCR4 cells, we implanted GL26-Cit-NT (N=10) and CXCR4 knock-down GL26-Cit-Sh2CXCR4 cells intracranially into Rag1^−/−^ mice (N=10). After tumor establishment, at day 10, animals (N=5) from both groups were given whole-brain IR (dose=2Gy/mouse/day) for the next 10 days. Thereafter, all four groups, (N=5/group) with and without radiation treatment were monitored until they became moribund (Figure 9A). Non-irradiated GL26-Cit-NT cells implanted mice began to succumb first to the tumors with a median survival of 20 days, while non-irradiated CXCR4 knock-down GL26-Cit-Sh2CXCR4 mice group exhibited a median survival of 26 days (Figure 9B and 9C). In the radiation-treated group, GL26-Cit-Sh2CXCR4 mice lived significantly longer with a median survival of 57 days while the control GL26-Cit-NT mice had a median survival time of 41 days. Thus, radiation treatment increased the survival of GL26-Cit-Sh2CXCR4 cells implanted by 39% after radiation treatment compared to GL26-Cit-NT mice (Figure 9B and 9C). GL26-Cit-Sh2CXCR4 cells implanted mice showed 130% increased survival with radiation treatment, when compared to non-irradiated mice implanted with same cells; whereas GL26-Cit-NT cells implanted mice exhibited increased survival of 100% with radiation treatment compared to non-radiated mice with control cells GL26-Cit-NT (Figure 9B and 9C). Together, our *in-vivo* studies confirmed that CXCR4 down regulated tumor-bearing mice survived longer than the control mice. Furthermore, combination of CXCR4 knock down with radiation treatment showed additional improvement in overall median survival (Figure 9B).

### CXCR4–deficient glioma cells undergo caspase-3–dependent cell death upon radiation treatment

**Figure 10:**
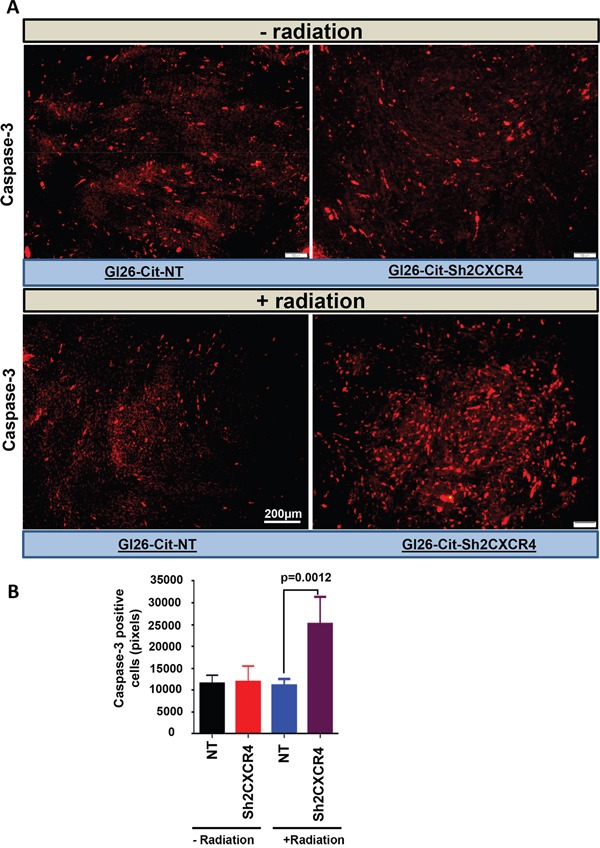
CXCR4–deficient glioma cells were more sensitive to radiation mediated cell death **A.** Brain section from untreated and radiation-treated mice were stained for cleaved-caspase-3 (1:300 dilution). Shown are representative sections from GL26-Cit-NT (left panel) and GL26-Cit-shCXCR4 (right panel). CXCR4 knock down tumor cells from radiated mice go through Caspase-3 mediated apoptosis **B.** Quantification of Caspase-3 staining from (A) comparing amount of apoptosis within GL26-Cit-NT and GL26-Cit-shCXCR4 after radiation treatment. (*p <0.0012, error bars represent SD).

To determine the role underlying the increased survival after radiation treatment in mice implanted with CXCR4 knockdown GL26-Cit-Sh2CXCR4 cells, we analyzed tumor sections from non-irradiated vs irradiated groups of mice for cleaved caspase-3, a major downstream effector mechanism for caspase-mediated apoptotic cell death. IHC data for caspase-3 staining revealed significantly larger numbers of GL26-Cit-Sh2CXCR4 cells undergoing apoptotic cell death upon radiation treatment compared to control to GL26-Cit-NT cells (Figure 10A). We further calculated the frequency of cleaved caspase-3 positive cells in all four tumor groups by using ImageJ software. CXCR4 knockdown GL26-Cit-Sh2CXCR4 tumors had significantly higher number of cleaved caspase-3 positive cells compared to GL26-Cit-NT. Increased cleaved caspase-3 expression in CXCR4-knocked down and irradiated tumors correlates with the increased survival of these mice (Figure 10B).

## DISCUSSION

Glioma cells invade the brain by active migration along blood vessels, white matter tracts, interstitially, or below the meninges. The highly invasive characteristic of GBMs is mainly responsible for the failure to control tumor growth after standard care like surgery, chemo- and radiation therapy. We and several other groups have described active migration of GBMs along the blood vessels perivascular space within the brain. Molecular mechanisms of the migration patterns of GBMs and the factors that attract glioma cells to perivascular space remain poorly understood. CXCR4 is expressed on different cells like neural and pluripotent stem cells and is important for directional migration during normal development. CXCR4 has also been reported to be expressed in metastasizing hepato-cellular, pancreatic, colorectal, cervical and melanoma cancer cells [36–38]. Zagzag et al depicted a correlation of CXCR4 expression on glioma cells with their localization in perineuronal, white matter, subpial and perivascular spaces. They also showed that blocking of CXCR4 with AMD3100 or with shRNA inhibits the migration of LN308 cells towards SDF-1 gradient *in-vitro*, but *in-vivo* effect of CXCR4 inhibiton on tumor invasion was not known [39]. AMD3100 mediated blockade of CXCR4 in U-118 glioma cells inhibiting their migration, chemotactic ability, and survival, albeit *in-vitro* [40]. Another study reported a direct tropic interaction of human glioma U87-Luc cells with CXCL12-expressing endothelial cells which was blocked by CXCR4 antagonists but not CXCR7 antagonists [41]. In a recent study, we showed that *in-vivo* CXCR4 blockade inhibits cell cycle progression, regulating survival and proliferation and also blocks the hypoxic-induction of HIF-1α and CXCL12 in GBM tumors generated using the Sleeping Beauty transposase method [42]. Thus, it may be likely that targeting CXCR4 offers therapeutic resistance to tumors. The current series of experiments tested hypothesis that the chemotactic migration of tumor cells in perivascular space and their resistance to cancer therapies depends on the expression of CXCR4 on glioma. Directional perivascular invasion and association of glioma cells to existing blood vasculature rely on active CXCR4 signaling and downregulation of CXCR4 will prevent perivascular invasion, and delay tumor growth.

To decipher the molecular mechanism involved in perivascular migration we used AMD3100 to block CXCR4 receptor on tumor cells and then measured their migration *in-vitro*. Here, we show that chemotactic factor/s secreted by MBVE or HBMVE cells induce directional migration of mouse and human glioma cells GL26-Cit and HF2303 *in-vitro*. CXCL12 appears to be the main factor regulating cell migration in this assay when cells are attracted by MBVE, given the inhibitory effectiveness of AMD3100. The lower capacity of AMD3100 to block migration towards HBMVE suggests that further molecules are involved in this process. These are currently being investigated. The invasion behavior of GL26-Cit and HF2303 glioma cells *in-vivo* was consistent with their migration patterns *in-vitro*; thus, using *in-vitro* migration models may serve as a good tool to identify molecular mechanisms responsible for glioma cell growth towards blood vessels. However, another human GBM cell line MGG8 did not show a directional migration towards blood vessels both *in-vitro* and *in-vivo*. This could be because they express lower CXCR4 compared to HF2303 as observed in Westen blot and micro-array data analysis. Alternatively may be because of their heterogeneous nature due to existence of different glioma stem-like cells, although they have not been separated into different groups yet. They are essentially characterized by their behavior, i.e., their capacity to grow as spheres, their capacity to differentiate into different neural cells, and their ability to form tumors *in-vivo* [3, 43, 44]. Thus, it would be possible that stem cells, just like other neural cells, could also be divided into subtypes. So far, however, this has not been proposed (or achieved). MGG8 cells are classed as glioma stem cells as they fulfill the three criteria mentioned above [45, 46].

AMD3100 was originally designed as a selective inhibitor of HIV-1 and HIV-2 replication [47]. It has been also used to inhibit autoimmune collagen-induced arthritis and several other diseases [31]. Although AMD3100 is shown to inhibit the interaction of CXCL12 and CXCR4, the specificity of AMD3100 for CXCR4 has never been extensively documented. Thus, off target effects cannot be ruled out. Although AMD3465 is another drug which is more specific than AMD3100 and has been shown to have anti-glioma effect in various models [48, 49], to rule out any unreported off target effects of both these drugs, we chose to selectively knock down CXCR4 expression using shRNA. Using this approach, we intended to specifically determine the role of CXCR4 in tumor migration and invasion of mouse glioma GL26-Cit cells towards endothelial cells. Migration of CXCR4 knockdown mouse glioma GL26-Cit cells was significantly reduced. Thus shRNA mediated selective knock down of CXCR4 demonstrates that CXCR4 plays critical role in chemotactic migration of GBM cells towards brain endothelial cells and downregulation of CXCR4 has direct impact on the ability of glioma cell migration.

Previous studies reported upregulation of CXCR4 expression in many GBM cell lines, as well as their responsiveness to vessel derived CXCL12 to stimulate their migration towards the vasculature [50, 51]. Thus, we anticipated that CXCR4-knockdown mouse glioma cells (GL26-Cit-Sh2CXCR4) will be less invasive due to reduced association with brain vessels. Indeed, GL26-Cit-sh2CXCR4 cells demonstrated significantly lower invasive capacity compared to control GL26-Cit-NT cells. Additionally, mice implanted with CXCR4 knock-down cells exhibit significantly increased survival compared to animals implanted with control cells. Low-CXCR4 expressing GL26-Cit-sh2CXCR4 cells have diminished association with blood vessels compared to CXCR4-sufficient GL26-Cit-NT implanted tumor. Thus, CXCR4 knockdown in glioma cells confirmed our hypothesis that glioma invasion of the perivascular space at the tumor border is dependent on CXCR4 expression on glioma cells. Thus reduction of perivascular space invasion is directly associated with increased survival.

Kioi et al demonstrated that expression of HIF-1 in tumors post irradiation recruits CXCR expressing BMDCs to restore the vasculature damage, thus aiding tumor growth and enhancing their resistance to further therapy [23]. We have shown earlier that in response to irradiation there is hypoxia associated with increased secretion of CXCL12 [42]. Since CXCL12 can increase tumor cell survival, knocking down CXCR4 would lead to an increase in glioma cell death. Our radiation experiment confirmed this hypothesis by showing increased apoptosis in glioma tumors knocked down for CXCR4 and irradiated. Thus, our experiments suggest that CXCR4 has a role in downregulating apoptosis, maintaining glioma cell viability, and thus reducing survival of irradiated glioma tumors.

Further, CXCR4-expression in tumors has been shown to correlate with perivascular migration, even though detailed study has not been performed so far [17, 50]. Emerging evidence indicates that the perivascular space is a specialized route for GBM invasion and it is proposed to enhance the resistance of glioma stem cells to radiation and chemotherapies by offering a niche [30]. It has also been shown that chemo- radiotherapy induces a CXCR4-dependent angiogenic switch which correlates to tumor recurrence. Our data demonstrates that in the absence of the CXCR4 receptor radiotherapy increases survival. Down regulating CXCR4 expression or blocking this receptor is a powerful mechanism for achieving anti-glioma resistance, further making the therapeutic effects of radiotherapy more evident [52]. If resistance to cancer therapies is dependent upon localization of tumor cells to the perivascular space, we asked if CXCR4-knock down glioma cells, which lose their ability to migrate into perivascular space would be more sensitive to radiation-mediated killing. In line with this, we observed that the extent of survival of mice intracranially implanted with low CXCR4-expressing GL26-Cit-sh2CXCR4 cells was significantly longer than mice implanted with control GL26-Cit cells. CXCR4 knock down cells were more sensitive to radiation- mediated killing as assessed by caspase-3 expression. Further, radiation treatment of GL26-Cit-sh2CXCR4 implanted mice increased the extent of survival when compared to all other groups tested. Studies have shown that post irradiation treatment, CXCR4 expression on glioma cells, including GL26 cells is upregulated suggesting a role of CXCR4 in tumor survival and therapeutic resistance. In this study, we show that a combined therapy of CXCR4 knock-down and radiation decreases the perivascular invasion of GBM and increases the sensitivity of tumor cells to radiation therapy. Our data agree with those of Liu et al who demonstrated in rats that inhibition of CXCL12 using NOX-A12, an l-enantiomeric RNA oligonucleotide, administered following tumor irradiation, caused tumor regression and prolonged survival compared to irradiation alone [53]. While use of AMD3100 for inhibition of CXCR4 interactions has been proposed [23], our data support a combined approach of using CXCR4 knock-down gene therapy with radiation therapy is more effective. This approach may provide a novel and powerful method for the treatment of malignant brain tumors.

## MATERIALS AND METHODS

### Animal strains

Six- to Eight-week-old female C57BL/6J, B6 and C57BL/6J, B6.129S7-Rag1tm1Mom/J (i.e., RAG1^−/−^), mice were purchased from Jackson Laboratory. Some C57BL6 mice were also purchased from tconic lab. All fully immunocompromised NSG mice were taken from breeding colony of Unit for Laboratory Animal Medicine (ULAM) at the University of Michigan. All animal experiments were conducted in accordance with procedures approved by the University Committee on Use and Care of Animals and conformed to the policies and procedures of the Unit for Laboratory Animal Medicine (ULAM) at the University of Michigan.

### Cell lines and primary cultures of human glioma stem cells

Mouse Gl26 glioma cell-line was obtained from the National Cancer Institute (Bethesda, MD). HF2303 primary human GBM cancer stem-cells were provided by Dr. Tom Mikkelsen, M.D. (Department of Neurology, Henry Ford Hospital, Detroit, MI). The MGG8 primary human GBM stem cells were obtained from Dr. Samuel Rabkin, Harvard University. Mouse Brain Microvascular Endothelial cells (MBVE) cells were purchased from Angio-Proteomics, Boston, MA and Human Brain Microvascular Endothelial (HBMVE) cells were provided by Dr. Anat Erdreich-Epstein (Children's Hospital of Los Angeles, Los Angeles, California). Mouse- (GL26) cells were genetically modified to express the fluorescent protein citrine, GL26-Cit, in order to visualize these cells at single cell resolution as described in our previous paper [54].

GL26-Cit and HF2303 primary human GBM cancer stem cells were cultured *in-vitro* under humidified conditions in 95% air 5% CO2 at 37°C. Culture medium composition for adherent glioma cells (i.e. GL26-Cit, HF2303, and MGG8) was Dulbecco's Modified Eagle Medium (DMEM) supplemented with 10% heat inactivated fetal bovine serum (FBS), 50μg/ml streptomycin, 0.3 mg/ml L-glutamine, 50U/ml penicillin 6μg/ml G418 selection antibiotic (for selection of the mCitrine expression vector). Cells were passaged every 2-4 days. Culture medium for primary human GBM cancer stem cells-derived neurospheres (HF2303 and MGG8) consisted of DMEM/F12 supplemented with N2 supplement (1x final concentration), and antibiotic-antimycotic reagent (0.5x final concentration). Fresh rEGF and rbFGF cytokines were added to neurosphere medium at each passage and every 3 days at a final concentration of 0.02μg/ml. HyClone HyQTase cell dissociation solution was used to split cells 1:3 every week. MBVE and HBMVE cells were gown as directed by manufacturer and HBMVE cells were cultured in RPMI1640 with 10% FBS, 2 mmol/L L-glutamine, 1 mmol/L sodium pyruvate, 20 mmol/L HEPES, 50 U/mL penicillin, and 50 mg/mL streptomycin.

### Antibodies

The following primary antibodies were used for staining: rat monoclonal anti-CD31/PECAM-1 (clone:MEC13.3), Cat#: 550274, BD Pharmingen; rabbit monoclonal anti-CXCR4, Cat# ab-124824, Abcam; chicken polyclonal anti-mouse nestin Cat#: N B100-1604, Novus biological. Cell signaling; rabbit polyclonal Cleaved Caspase-3 (Asp175) Antibody Cat#: 9661.

### Transwell migration assay

Transwell migration assays were performed using 8.0 μm pore-size Transwell assay (Corning Inc, Corning, NY, USA) and Transwells were built within a 24-well plate (Costar). 100,000 mouse brain-derived vascular endothelial (MBVE) cells or human brain microvasculature endothelial cells (HBMVE) cells were seeded on bottom chambers of trans-well in 650 μL of DMEM plus 10% FBS medium. Plated cells were incubated in humidified incubator (37°C, 5% CO2) for 24 hours. Next day 50,000 tumor cells (GL26-Cit, HF2303, or MGG8) were added to the transwell that was in contact with the medium at the bottom of the well. 12 hours later, glioma cells that migrated to the lower side of transwell insert were tripsinized (0.05% trypsin) and counted with a hemocytometer.

For AMD3100 inhibition assay, 100,000 MBVE or HBMVE cells were plated as described in above section. After 24 hours, MBVE or HBMVE and 50,000 tumor cells were treated with AMD3100 (10 μM) or control vehicle for 1 hour prior to transwell incubation. Migrated cells were counted with hemocytometer after trypsinization.

### Generation of CXCR4 downregulated GL26-Cit cells using shRNA

To knockdown CXCR4 in GL26-Cit cells, pLKO.1-puro lentiviral plasmids encoding four different shRNA hairpins constructs specific for mouse CXCR4 (NM_009911) along with puromycin resistance cassette were purchased from Sigma Aldrich as part of the mission shRNA Consortium. Each shRNA clone was tested for its' ability to knockdown CXCR4 expression. Second-generation lentiviruses encoding two shRNA constructs (TRCN0000028704, CXCR4 shRNA1 and TRCN0000028749, CXCR4 shRNA2) were prepared according to manufacturer's instructions (Clonetech_LentiXTX transfection kit). (Cat# 631317). 5 x106 HEK293TX cells were seeded 24 hours prior to transfection with each of the shRNA construct along with Lenti-X HTX packaging mix, then cells were incubated for 48 hours at 37°C. Lentiviral particles were purified from culture supernatant. GL26-Cit cells were infected with purified mouse CXCR4–specific shRNA lentivirus for 48-72 hours and subjected to puromycin selection for 2 weeks. A western blot analysis was performed to validate stable CXCR4 knockdown in GL26-Cit cell line. Similar methodology was used to create GL26-Cit-NT control cell-line, which consisted pLKO.1-puro lentiviral expression vector and non-targeting shRNA hairpin construct (Mission shRNA, Cat#:SHC002, Sigma-Aldrich).

### Western blot

Whole cell extracts were prepared by lysing the cells with RIPA buffer (50 mM Tris, pH-7.4; 150 mM NaCl; 1 mM each NaF, NaVO4, and EGTA; 1% Igepol; 0.25% sodium deoxycholate; 1x protease/phosphotase inhibitors (Thermo Scientific, Waltham, MA) and placed on ice for 5 min. After incubation, lysates were collected and centrifuged at 1,300 RPM for 10min at 4°C to exclude cell debris. Protein supernatants were collected. Protein concentration was measured with Pierce BCA Protein Assay Kit (Thermo Scientific). Cell lysates were either stored at -80°C or used immediately for SDS-PAGE/Western blot analysis. For SDS-PAGE, 30 μg total proteins were reduced and denatured at 95°C for 10min and loaded onto a 4-12% SDS polyacrylamide gradient gel to run at 200V until sufficient protein separation was achieved. Protein samples were transferred onto PVDF membranes for immunoblotting for ~100min at 30V in ice-cold transfer buffer. PVDF membrane was blocked with 5% milk for 30 to 45 min and incubated with primary antibodies as per manufactures suggested dilution overnight at 4°C. Membranes were washed 3 times in TBS with 0.1% Tween20 and incubated with appropriate HRP-conjugated secondary antibodies (Dako, Carpinteria, CA) for 1hr. PVDF membrane was washed again 3 times with TBS+0.1% Tween20. Immunoreactivity was visualized using the SuperSignal West Femto substrate solution (Thermo Scientific) and imagedon a ChemiDoc MP imaging system (BioRad, Hercules, CA).

### Stereotactic tumor implantation and processing of tissue samples

Six- to eight-week-old female or male mice received a single i.p. injection of 75 mg/kg ketamine and 0.5 mg/kg dexmedetomidine in sterile 0.9% NaCl prior to surgery. Once anesthetized, the rodents were placed into a stereotactic frame. A single midline incision was made from the frontal bone, caudally, to expose the cranium. Then a single hole was drilled into the cranium above the left cerebral hemisphere, using a precision power drill equipped with a fine bit at +0.5mm AP,+2.5mm ML, and −3.0mm DV from the bregma. Upon reaching the dura, 1x103 cells in 1μL of serum free DMEM were implanted with a Hamilton syringe equipped with a 33-gauge needle. Cells were allowed to settle for 5min followed by slow needle withdrawal. The skin was then sutured and animals were treated with anesthesia reversal and post-operative pain relief respectively with doses of atipamezole (1 mg/kg i.m.) and buprenorphine (0.1 mg/kg s.c.). Animals underwent transcardial perfusion either at predetermined time-points post tumor implantation or once reaching a moribund state. Prior to perfusion, animals were injected i.p. with 50 mg/kg of both ketamine and xylazine to induce deep anesthesia. Animals were transcardially perfused with oxygenated and heparinized (100U/L) Tyrode's solution using a peristaltic pump followed by 4% paraformaldehyde (PFA) pH7.4 in PBS. Brains were collected and stored in 4% PFA at 4°C, in the dark, prior to sectioning 50μm thick coronal tissue via vibratome for immunolabeling and/or microscopic analysis.

### Immunohistochemistry

Brains were coronally sectioned every 50μm in a serial fashion using a Vibratome and stored in 12-well plates containing PBS-0.1% sodium azide at 4°C in the dark. Brain sections chosen for immunolabeling were placed into a 12-well plate containing TBS-0.1% Triton-X (TBS-Tx) for 60min. When antigen retrieval was required, TBS-Tx was replaced with boiling 10mM sodium citrate solution. Non-specific antibody binding was blocked with 10% normal goat serum in TBS-Tx for 1hr incubation at room temperature. Sections were then transferred to primary antibody solution diluted in TBS-Tx containing 1% NGS and 0.1% sodium azide for 24-48hrs at room temperature, in the dark. Sections were washed 6 times in TBS-Tx and incubated in secondary antibody solution containing 1% NGS in TBS-Tx for 12-24hrs at room temperature, in the dark. Finally, sections were washed 6 times with TBS-Tx followed by incubation with a stock solution of DAPI [5 mg/ml] diluted [1:1,000] in PBS for 6min. Sections were washed again 3 time in PBS, mounted on microscope slides, and the cover slip was applied using prolong gold anti-fade reagent. During all incubation and washing steps, the 12-plate was placed on a shaker.

### Statistical analysis

Statistical analyses were performed using GraphPad Prism 7 (GraphPad Software, Inc., La Jolla, CA). Data are represented as mean ±SEM and were examined with the statistical tests specified in each figure legend. Values were considered significant at the P ≤.05 level.

## References

[1] Scherer HJ (1940). A Critical Review: The Pathology of Cerebral Gliomas. J Neurol Psychiatry.

[2] Wen PY, Kesari S (2008). Malignant gliomas in adults. N Engl J Med.

[3] Singh SK, Hawkins C, Clarke ID, Squire JA, Bayani J, Hide T, Henkelman RM, Cusimano MD, Dirks PB (2004). Identification of human brain tumour initiating cells. Nature.

[4] Baker GJ, Yadav VN, Motsch S, Koschmann C, Calinescu AA, Mineharu Y, Camelo-Piragua SI, Orringer D, Bannykh S, Nichols WS, deCarvalho AC, Mikkelsen T, Castro MG (2014). Mechanisms of glioma formation: iterative perivascular glioma growth and invasion leads to tumor progression, VEGF-independent vascularization, and resistance to antiangiogenic therapy. Neoplasia.

[5] Ries LAG, Kosary CL, Hankey BF, Miller BA, Clegg L, Edwards BK (2001). SEER Cancer Statistics Review, 1973-1998. National Cancer Institute Bethesda.

[6] Ramirez YP, Weatherbee JL, Wheelhouse RT, Ross AH (2013). Glioblastoma multiforme therapy and mechanisms of resistance. Pharmaceuticals (Basel).

[7] Altaner C, Altanerova V (2012). Stem cell based glioblastoma gene therapy. Neoplasma.

[8] Ping YF, Bian XW (2011). Consice review: Contribution of cancer stem cells to neovascularization. Stem Cells.

[9] Sakariassen PO, Immervoll H, Chekenya M (2007). Cancer stem cells as mediators of treatment resistance in brain tumors: status and controversies. Neoplasia.

[10] Winkler F, Kienast Y, Fuhrmann M, Von Baumgarten L, Burgold S, Mitteregger G, Kretzschmar H, Herms J (2009). Imaging glioma cell invasion *in vivo* reveals mechanisms of dissemination and peritumoral angiogenesis. Glia.

[11] Farin A, Suzuki SO, Weiker M, Goldman JE, Bruce JN, Canoll P (2006). Transplanted glioma cells migrate and proliferate on host brain vasculature: a dynamic analysis. Glia.

[12] Holash J, Maisonpierre PC, Compton D, Boland P, Alexander CR, Zagzag D, Yancopoulos GD, Wiegand SJ (1999). Vessel cooption, regression, and growth in tumors mediated by angiopoietins and VEGF. Science.

[13] Watkins S, Robel S, Kimbrough IF, Robert SM, Ellis-Davies G, Sontheimer H (2014). Disruption of astrocyte-vascular coupling and the blood-brain barrier by invading glioma cells. Nat Commun.

[14] Zagzag D, Krishnamachary B, Yee H, Okuyama H, Chiriboga L, Ali MA, Melamed J, Semenza GL (2005). Stromal cell-derived factor-1alpha and CXCR4 expression in hemangioblastoma and clear cell-renal cell carcinoma: von Hippel-Lindau loss-of-function induces expression of a ligand and its receptor. Cancer Res.

[15] Sun YX, Wang J, Shelburne CE, Lopatin DE, Chinnaiyan AM, Rubin MA, Pienta KJ, Taichman RS (2003). Expression of CXCR4 and CXCL12 (SDF-1) in human prostate cancers (PCa) *in vivo*. J Cell Biochem.

[16] Kato M, Kitayama J, Kazama S, Nagawa H (2003). Expression pattern of CXC chemokine receptor-4 is correlated with lymph node metastasis in human invasive ductal carcinoma. Breast Cancer Research.

[17] Ehtesham M, Winston JA, Kabos P, Thompson RC (2006). CXCR4 expression mediates glioma cell invasiveness. Oncogene.

[18] Kijima T, Maulik G, Ma PC, Tibaldi EV, Turner RE, Rollins B, Sattler M, Johnson BE, Salgia R (2002). Regulation of cellular proliferation, cytoskeletal function, and signal transduction through CXCR4 and c-Kit in small cell lung cancer cells. Cancer Res.

[19] Ehtesham M, Mapara KY, Stevenson CB, Thompson RC (2009). CXCR4 mediates the proliferation of glioblastoma progenitor cells. Cancer Lett.

[20] Lee CC, Lai JH, Hueng DY, Ma HI, Chung Y, Sun YY, Tsai YJ, Wu WB, Chen CL (2013). Disrupting the CXCL12/CXCR4 axis disturbs the characteristics of glioblastoma stem-like cells of rat RG2 glioblastoma. Cancer Cell Int.

[21] Schulte A, Gunther HS, Phillips HS, Kemming D, Martens T, Kharbanda S, Soriano RH, Modrusan Z, Zapf S, Westphal M, Lamszus K (2011). A distinct subset of glioma cell lines with stem cell-like properties reflects the transcriptional phenotype of glioblastomas and overexpresses CXCR4 as therapeutic target. Glia.

[22] Gatti M, Pattarozzi A, Bajetto A, Wurth R, Daga A, Fiaschi P, Zona G, Florio T, Barbieri F (2013). Inhibition of CXCL12/CXCR4 autocrine/paracrine loop reduces viability of human glioblastoma stem-like cells affecting self-renewal activity. Toxicology.

[23] Kioi M, Vogel H, Schultz G, Hoffman RM, Harsh GR, Brown JM (2010). Inhibition of vasculogenesis, but not angiogenesis, prevents the recurrence of glioblastoma after irradiation in mice. J Clin Invest.

[24] Wang SC, Yu CF, Hong JH, Tsai CS, Chiang CS (2013). Radiation therapy-induced tumor invasiveness is associated with SDF-1-regulated macrophage mobilization and vasculogenesis. PLoS One.

[25] Goffart N, Lombard A, Lallemand F, Kroonen J, Nassen J, Di Valentin E, Berendsen S, Dedobbeleer M, Willems E, Robe P, Bours V, Martin D, Martinive P (2016). CXCL12 mediates glioblastoma resistance to radiotherapy in the subventricular zone. Neuro Oncol.

[26] Barone A, Sengupta R, Warrington NM, Smith E, Wen PY, Brekken RA, Romagnoli B, Douglas G, Chevalier E, Bauer MP, Dembowsky K, Piwnica-Worms D, Rubin JB (2014). Combined VEGF and CXCR4 antagonism targets the GBM stem cell population and synergistically improves survival in an intracranial mouse model of glioblastoma. Oncotarget.

[27] Walters MJ, Ebsworth K, Berahovich RD, Penfold ME, Liu SC, Al Omran R, Kioi M, Chernikova SB, Tseng D, Mulkearns-Hubert EE, Sinyuk M, Ransohoff RM, Lathia JD (2014). Inhibition of CXCR7 extends survival following irradiation of brain tumours in mice and rats. Br J Cancer.

[28] Redjal N, Chan JA, Segal RA, Kung AL (2006). CXCR4 inhibition synergizes with cytotoxic chemotherapy in gliomas. Clin Cancer Res.

[29] Rubin JB, Kung AL, Klein RS, Chan JA, Sun Y, Schmidt K, Kieran MW, Luster AD, Segal RA (2003). A small-molecule antagonist of CXCR4 inhibits intracranial growth of primary brain tumors. Proc Natl Acad Sci U S A.

[30] Wurth R, Bajetto A, Harrison JK, Barbieri F, Florio T (2014). CXCL12 modulation of CXCR4 and CXCR7 activity in human glioblastoma stem-like cells and regulation of the tumor microenvironment. Front Cell Neurosci.

[31] De Clercq E (2003). The bicyclam AMD3100 story. Nat Rev Drug Discov.

[32] De Clercq E (2009). The AMD3100 story: the path to the discovery of a stem cell mobilizer (Mozobil). Biochem Pharmacol.

[33] Kalatskaya I, Berchiche YA, Gravel S, Limberg BJ, Rosenbaum JS, Heveker N (2009). AMD3100 is a CXCR7 ligand with allosteric agonist properties. Mol Pharmacol.

[34] Uto-Konomi A, McKibben B, Wirtz J, Sato Y, Takano A, Nanki T, Suzuki S (2013). CXCR7 agonists inhibit the function of CXCL12 by down-regulation of CXCR4. Biochem Biophys Res Commun.

[35] Walter HL, van der Maten G, Antunes AR, Wieloch T, Ruscher K (2015). Treatment with AMD3100 attenuates the microglial response and improves outcome after experimental stroke. J Neuroinflammation.

[36] Scala S, Giuliano P, Ascierto PA, Ierano C, Franco R, Napolitano M, Ottaiano A, Lombardi ML, Luongo M, Simeone E, Castiglia D, Mauro F, De Michele I (2006). Human melanoma metastases express functional CXCR4. Clin Cancer Res.

[37] Schimanski CC, Schwald S, Simiantonaki N, Jayasinghe C, Gonner U, Wilsberg V, Junginger T, Berger MR, Galle PR, Moehler M (2005). Effect of chemokine receptors CXCR4 and CCR7 on the metastatic behavior of human colorectal cancer. Clin Cancer Res.

[38] Koshiba T, Hosotani R, Miyamoto Y, Ida J, Tsuji S, Nakajima S, Kawaguchi M, Kobayashi H, Doi R, Hori T, Fujii N, Imamura M (2000). Expression of stromal cell-derived factor 1 and CXCR4 ligand receptor system in pancreatic cancer: a possible role for tumor progression. Clin Cancer Res.

[39] Zagzag D, Esencay M, Mendez O, Yee H, Smirnova I, Huang Y, Chiriboga L, Lukyanov E, Liu M, Newcomb EW (2008). Hypoxia- and vascular endothelial growth factor-induced stromal cell-derived factor-1alpha/CXCR4 expression in glioblastomas: one plausible explanation of Scherer's structures. Am J Pathol.

[40] do Carmo A, Patricio I, Cruz MT, Carvalheiro H, Oliveira CR, Lopes MC (2010). CXCL12/CXCR4 promotes motility and proliferation of glioma cells. Cancer Biol Ther.

[41] Rao S, Sengupta R, Choe EJ, Woerner BM, Jackson E, Sun T, Leonard J, Piwnica-Worms D, Rubin JB (2012). CXCL12 mediates trophic interactions between endothelial and tumor cells in glioblastoma. PLoS One.

[42] Calinescu AA, Yadav VN, Carballo E, Kadiyala P, Tran D, Zamler D, Doherty R, Srikanth M, Lowenstein PR, Castro MG (2016). Survival and proliferation of neural progenitor derived glioblastomas under hypoxic stress is controlled by a CXCL12/CXCR4 autocrine positive feedback mechanism. Clin Cancer Res.

[43] Galli R, Binda E, Orfanelli U, Cipelletti B, Gritti A, De Vitis S, Fiocco R, Foroni C, Dimeco F, Vescovi A (2004). Isolation and characterization of tumorigenic, stem-like neural precursors from human glioblastoma. Cancer Res.

[44] Beier D, Hau P, Proescholdt M, Lohmeier A, Wischhusen J, Oefner PJ, Aigner L, Brawanski A, Bogdahn U, Beier CP (2007). CD133(+) and CD133(-) glioblastoma-derived cancer stem cells show differential growth characteristics and molecular profiles. Cancer Res.

[45] Wilson TJ, Zamler DB, Doherty R, Castro MG, Lowenstein PR (2016). Reversibility of glioma stem cells' phenotypes explains their complex *in vitro* and *in vivo* behavior. Discovery of a novel neurosphere-specific enzyme, cGMP-dependent protein kinase 1, using the genomic landscape of human glioma stem cells. Oncotarget.

[46] Suva ML, Rheinbay E, Gillespie SM, Patel AP, Wakimoto H, Rabkin SD, Riggi N, Chi AS, Cahill DP, Nahed BV, Curry WT, Martuza RL, Rivera MN (2014). Reconstructing and reprogramming the tumor-propagating potential of glioblastoma stem-like cells. Cell.

[47] De Clercq E, Yamamoto N, Pauwels R, Baba M, Schols D, Nakashima H, Balzarini J, Debyser Z, Murrer BA, Schwartz D (1992). Potent and selective inhibition of human immunodeficiency virus (HIV)-1 and HIV-2 replication by a class of bicyclams interacting with a viral uncoating event. Proc Natl Acad Sci U S A.

[48] Yang L, Jackson E, Woerner BM, Perry A, Piwnica-Worms D, Rubin JB (2007). Blocking CXCR4-mediated cyclic AMP suppression inhibits brain tumor growth *in vivo*. Cancer Res.

[49] Smith MC, Luker KE, Garbow JR, Prior JL, Jackson E, Piwnica-Worms D, Luker GD (2004). CXCR4 regulates growth of both primary and metastatic breast cancer. Cancer Res.

[50] Zagzag D, Lukyanov Y, Lan L, Ali MA, Esencay M, Mendez O, Yee H, Voura EB, Newcomb EW (2006). Hypoxia-inducible factor 1 and VEGF upregulate CXCR4 in glioblastoma: implications for angiogenesis and glioma cell invasion. Lab Invest.

[51] Hong X, Jiang F, Kalkanis SN, Zhang ZG, Zhang XP, DeCarvalho AC, Katakowski M, Bobbitt K, Mikkelsen T, Chopp M (2006). SDF-1 and CXCR4 are up-regulated by VEGF and contribute to glioma cell invasion. Cancer Lett.

[52] Tabouret E, Tchoghandjian A, Denicolai E, Delfino C, Metellus P, Graillon T, Boucard C, Nanni I, Padovani L, Ouafik L, Figarella-Branger D, Chinot O (2015). Recurrence of glioblastoma after radio-chemotherapy is associated with an angiogenic switch to the CXCL12-CXCR4 pathway. Oncotarget.

[53] Liu SC, Alomran R, Chernikova SB, Lartey F, Stafford J, Jang T, Merchant M, Zboralski D, Zollner S, Kruschinski A, Klussmann S, Recht L, Brown JM (2014). Blockade of SDF-1 after irradiation inhibits tumor recurrences of autochthonous brain tumors in rats. Neuro Oncol.

[54] Baker GJ, Chockley P, Yadav VN, Doherty R, Ritt M, Sivaramakrishnan S, Castro MG, Lowenstein PR (2014). Natural killer cells eradicate galectin-1-deficient glioma in the absence of adaptive immunity. Cancer Res.

